# A systematic review of treatment attrition in anorexia nervosa

**DOI:** 10.1186/2050-2974-1-S1-P1

**Published:** 2013-11-14

**Authors:** Ghafda Abdelbaky, Philipa Hay, Stephen Touyz

**Affiliations:** 1 Institute of Psychiatry Fellow, School of Medicine, University of Western Sydney, Australia; 2 School of Medicine, University of Western Sydney, Australia; 3 School of Psychology and Centre for Eating and Dieting Disorders (Boden Institute), University of Sydney, Australia

## Background

Understanding of reasons for attrition in anorexia nervosa therapy is incomplete.

## Aims

This systematic review was of trials that reported factors associated with attrition, and aimed to compare and contrast findings between treatment settings.

## Methods

Data were extracted from published reports sourced from searches (dates to 2/2013) of SCOPUS, PubMED, PsycINFO, included French and English language papers, and search terms: 'anorexia nervosa' and 'attrition /drop-out/premature termination of treatment/outcome'.

## Results

421 papers were identified, 34 met inclusion criteria, 4 were excluded as they were reviews, and 3 investigated outcome not attrition. Two papers of the 27 included were qualitative studies. Factors consistently associated with attrition in any treatment setting were the type of anorexia nervosa, where the purging type was associated with higher attrition rate than the restrictive type, and poor motivation to change. Less consistent findings were reported in regards to co-morbidity and personality features. The majority of trials were of adults or older adolescents, and over 70% were of inpatient samples.

## Conclusion

More studies of attrition in younger people and outpatient settings, and more consistent and standardised assessment of co-morbidity and personality in anorexia nervosa research is needed. Expanding motivational enhancement strategies in therapy may reduce attrition.

